# Fungal Secondary Metabolites/Dicationic Pyridinium Iodide Combinations in Combat against Multi-Drug Resistant Microorganisms

**DOI:** 10.3390/molecules28062434

**Published:** 2023-03-07

**Authors:** Ayoub M. Abdelalatif, Bassma H. Elwakil, Mohamed Zakaria Mohamed, Mohamed Hagar, Zakia A. Olama

**Affiliations:** 1 Department of Botany & Microbiology, Faculty of Science, Alexandria University, Alexandria 21568, Egypt; 2 Department of Medical Laboratory Technology, Faculty of Applied Health Sciences Technology, Pharos University in Alexandria, Alexandria 21526, Egypt; 3 Department of Chemistry, Faculty of Science, Alexandria University, Alexandria 21568, Egypt

**Keywords:** endophytic fungi, *Trichoderma lixii*, *Aspergillus niger*, dicationic pyridinium iodide, multi-drug resistant

## Abstract

The spread of antibiotic-resistant opportunistic microbes is a huge socioeconomic burden and a growing concern for global public health. In the current study, two endophytic fungal strains were isolated from *Mangifera Indica* roots and identified as *Aspergillus niger* MT597434.1 and *Trichoderma lixii* KU324798.1. Secondary metabolites produced by *A. niger* and *T. lixii* were extracted and tested for their antimicrobial activity. The highest activity was noticed against *Staphylococcus aureus* and *E. coli* treated with *A. niger* and *T. lixii* secondary metabolites, respectively. *A. niger* crude extract was mainly composed of Pentadecanoic acid, 14-methyl-, methyl ester and 9-Octadecenoic acid (***Z***)-, methyl ester (26.66 and 18.01%, respectively), while *T. lixii* crude extract’s major components were 2,4-Decadienal, (*E*,*E*) and 9-Octadecenoic acid (*Z*)-, and methyl ester (10.69 and 10.32%, respectively). Moreover, a comparative study between the fungal extracts and dicationic pyridinium iodide showed that the combination of *A. niger* and *T. lixii* secondary metabolites with dicationic pyridinium iodide compound showed a synergistic effect against *Klebsiella pneumoniae*. The combined formulae inhibited the bacterial growth after 4 to 6 h through cell wall breakage and cells deformation, with intracellular components leakage and increased ROS production.

## 1. Introduction

The emergence of multidrug-resistant microorganisms has increased the urgency of finding effective new antimicrobials to treat bacterial, fungal, and viral illnesses in humans and animals [1]. β-lactam resistance emergence in Gram-negative bacteria has been a major concern that has become an obstacle in the treatment of infectious diseases, especially those caused by *Klebsiella pneumoniae*. Miryala et al. [2] studied the role of the SHV-11 gene in drug resistance mechanism patterns in a *K. pneumoniae* strain. It was concluded that the SHV11 gene, along with the functional partners, were not only responsible for the drug resistance mechanism, but also helped in maintaining the genomic integrity through the DNA damage repair mechanism.

On the other hand, endophytic fungi are a broad collection of microorganisms that live either entirely or partially inside the cells of their host plants, invading healthy tissues with no outward sign of illness [3]. More and more chemicals with diverse biological functions are being extracted from endophytic fungi [4]. Natural products with biological functions which are called secondary metabolites and endophytic filamentous fungi are among the most prolific producers [4]. Many useful bioactive chemicals with antibacterial, insecticidal, cytotoxic, and anticancer activities have been isolated in the last two decades from endophytic fungi [5]. Alkaloids, terpenoids, steroids, quinolones, isocoumarins, lignans, phenylpropanoids, phenols, and lactones are some of the most common classes of fungal bioactive chemicals [6]. Antimicrobial action against a wide variety of microorganisms has been shown by *Trichoderma* sp., a fungal species present in many habitats [7]. Moreover, *Aspergillus niger* is one of the most well-known fungi, and has been isolated from several niches (soil, nuts and food). Extracellular enzymes and citric acid produced from *A. niger* are known as Generally Recognized As Safe for human consumption (GRAS) by the FDA because of their usage in several industrial settings [8]. Hence, *A. niger* has been considered a valuable resource for the biotechnological sector due to the abundance of secondary metabolites with immunomodulatory and cytotoxic properties against cancer cells [9].

On the other hand, chemically synthesized compounds, namely ionic liquids (ILs), are one of the most interesting scientific and technological advancements for their various applications over the last few decades. There have been significant developments regarding the relevance of these types of unique molecules with adjustable biological and industrial properties [10,11]. Initially, ionic liquids were identified as a combination of inorganic counter anions and organic counter cations. During the synthesis of ionic liquids, the generation of nitrogen-containing heterocyclic molecules contributes significantly [12]. In contrast, hydrazones have become significant molecules in modern chemical synthesis, garnering considerable interest. They were used in a variety of pharmaceuticals and chemotherapeutic drugs [13]. Their attachment to organic molecules plays a crucial role in essential biological processes and in the formulation of medications with a wide range of biological characteristics, including antibacterial [14], anticancer [15], anti-inflammatory [16], antifungal [17], and antitubercular [18] activities. Recently, dicationic ionic liquids (DiILs), a new category of the ILs family, has attracted a great amount of researchers’ attention as it represents an interesting variation of the cationic partner. DiILs consist of two head groups (cations) linked by a rigid or flexible spacer and two anions [19].

Hence, the aim of the present study was to synthesize a dicationic pyridinium iodide compound, and characterize and combine it with a biologically active natural product for its potential synergistic effect. 

## 2. Results and Discussions

### 2.1. Molecular Identification of Fungal Isolates

**Figure 1:**
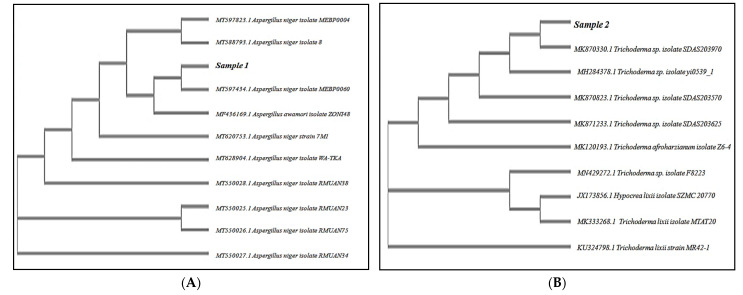
Phylogenetic tree of *A. niger* MT597434.1 (**A**) and *Trichoderma lixii* KU324798.1 (**B**).

In the current study, two endophytic fungal strains were isolated from *Mangifera Indica* roots. The isolates were identified using ITS4 and ITS5 rRNA sequencing. The sequences obtained were compared with the nucleotide sequences of the international database. The isolated fungal strains were *Aspergillus niger* with GenBank accession number MT597434.1 (100% similarity) and *Trichoderma lixii* with GenBank accession number KU324798.1 (98.18% similarity). Furthermore, the phylogenetic tree was generated by performing a distance matrix analysis (Figure 1).

### 2.2. Antibacterial Activity of Fungal Bioactive Secondary Metabolites

Data in Table 1 revealed that the inhibition zones (IZ) diameter of *T. lixii* and *A. niger* crude extracts ranged from 8.0 to 20.0 mm and from 7.5 to 21.0 mm, respectively, against the tested pathogens. *Staphylococcus aureus* and *E. coli* were the most susceptible organisms against *A. niger* and *T. lixii* crude extracts, respectively.

Quang et al. [20] stated that *A. niger* metabolites have been considered as a promising source of antibiotics that inhibit the growth of the Gram-positive bacterium *E. faecalis*, with MIC values ranging from 32 to 64 mM, and of *Candida albicans*, with MIC values ranged from 64 to 128 mM. Meanwhile, Padhi et al. [21] revealed that *A. niger* metabolites showed antifungal activity against *Candida albicans* with IC_50_ 31 mg/mL, and antibacterial activity against *Pseudomonas aeruginosa*, *Escherichia coli* and *Staphylococcus aureus* with IC_50_ of 160 mg/mL, 47 mg/mL and 135 mg/mL, respectively. Chigozie et al. [22] reported that the fungal extract of *Aspergillus* sp. isolated from fresh leaves of *Mangifera indica*. exhibited antibacterial activity against *P. aeruginosa* and *E. coli*.

### 2.3. GC-MS Analysis of Fungal Secondary Metabolites

**Figure 2:**
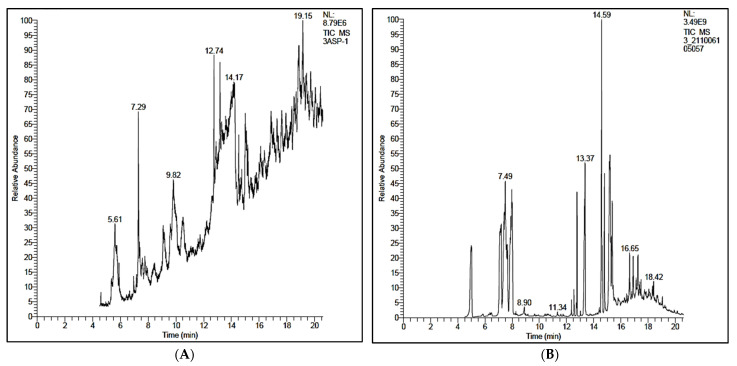
GC-MS chromatogram of *Aspergillus niger* (**A**), and *Trichoderma lixii* (**B**).

Data in Figure 2 proved that the *A. niger* crude extract was mainly composed of Pentadecanoic acid, 14-methyl-, methyl ester and 9-Octadecenoic acid (*Z*)-, and methyl ester (26.66 and 18.01%, respectively). However, the *T. lixii* crude extract’s relatively major components were 2,4-Decadienal, (*E*,*E*) and 9-Octadecenoic acid (*Z*)-, and methyl ester (10.69 and 10.32%, respectively) (Table 2). Venice et al. [23] stated that a GC-MS analysis of *T. lixii* crude extract identified the presence of 1,3,3-Trimethyl-Diepoxyhexadecane and 3-Octadecenoic acid compounds. An analysis of endophytes’ diversity has determined relationships among host plants and the endophytic fungi, through determining various secondary metabolites biosynthesized from the culture extract of the endophytic fungal isolates [23].

### 2.4. Molecular Docking Study

Among the most common mechanisms, the Extended-spectrum β-lactamases (ESBLs) were widely reported [24]. One of the main concerns is that resistance caused by these enzymes may result in an efficacy reduction of antimicrobial therapy, or in failed treatment [25]. The reported findings demonstrated that ESBL-variants of SHV-type were the most frequent mechanisms of resistance in ESBL-producing *K. pneumoniae* isolates implicated in bacteremia. Hence, the SHV enzyme was chosen in the present investigation to assess the possible mechanistic action of the synthesized dicationic pyridinium iodide compound, as well as the naturally extracted compounds.

In the current study, molecular docking was performed to predict the binding affinity of the naturally extracted and chemically synthesized compounds toward the target ESBL enzyme SHV-1 (Table 3). The results of the docking studies showed an excellent binding manner with the active site of the target macromolecules, in comparison to the reference drug co-crystallized ligand LN1-255. The naturally extracted compounds showed higher binding scores when compared to the dicationic pyridinium iodide compound (−6.38 kcal/mol), where Pentadecanoic acid, 14-methyl-, methyl ester and 9-Octadecenoic acid (*Z*)-, methyl ester (extracted from *Aspergillus niger*) had −6.51 and −6.50 kcal/mol. *Trichoderma lixii*’s most potent compounds were 9-Octadecenoic acid (*Z*)-, methyl ester, 1,2-15,16-Diepoxyhexadecane and Heptadecane, 9hexyl, showing −6.96, −6.56 and −6.99 kcal/mol binding affinity, respectively. The binding interactions of the dicationic pyridinium iodide compound revealed that the dicationic pyridinium iodide compound was well oriented inside the enzyme pockets and showed hydrophobic interaction with Arg244, Ala280 and Tyr105.

### 2.5. Comparative Study between T. lixii, A. niger Crude Extracts and Dicationic Pyridinium Iodide Compound

#### 2.5.1. Disc Diffusion Technique

**Figure 3:**
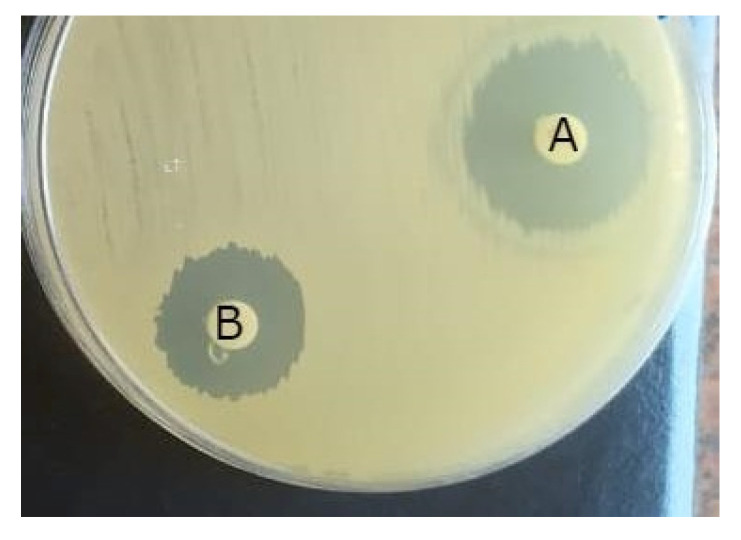
The combined effect of dicationic pyridinium iodide compound with *T. lixii* crude extracts (A) and *A. niger* (B) against *K. pneumoniae*.

Data in Table 4 revealed that the combined action of *A. niger* crude extract with the dicationic pyridinium iodide compound was synergistic against all the tested pathogens except *S. aureus*, while the combined action of *T. lixii* crude extract with dicationic pyridinium iodide was synergistic only against *K. pneumoniae* (Figure 3). Hence, *K. pneumoniae* was selected for further analyses.

#### 2.5.2. Checkerboard Dilution Technique

Data in Table 5 proved that the combined actions of the dicationic pyridinium iodide compound with *A. niger* and *T. lixii* crude extracts were synergistic against *K. pneumoniae*, with FICI 0.35 and 0.4, respectively. The observed antibacterial effect was further investigated against *K. pneumoniae* based on the FICI and MIC values.

### 2.6. Mechanistic Action of the Combined Formula

**Figure 4:**
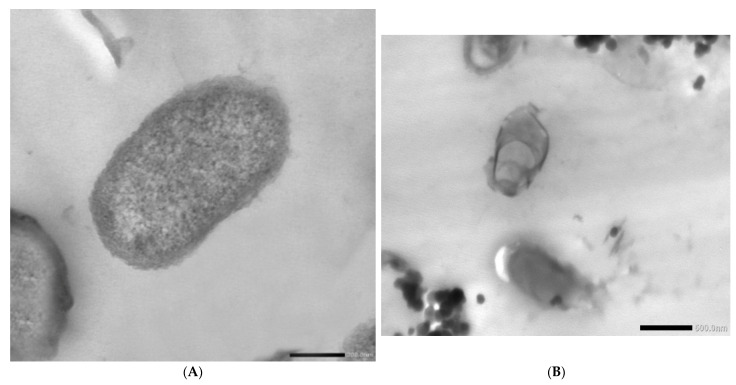

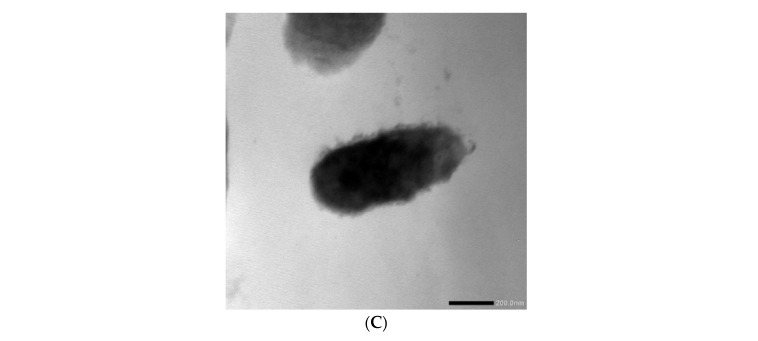
Transmission electron microscopic micrograph of *K. pneumoniae* untreated control (**A**), *T. Lixii*/dicationic pyridinium iodide (**B**) and *A. niger*/dicationic pyridinium iodide (**C**) treated cells.

**Figure 5:**
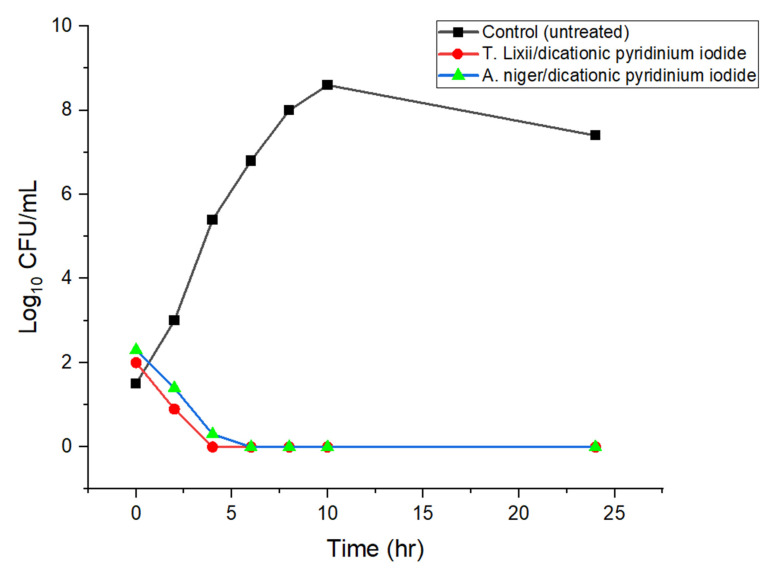
Bacterial growth in the presence and absence of the combined formulae.

**Figure 6:**
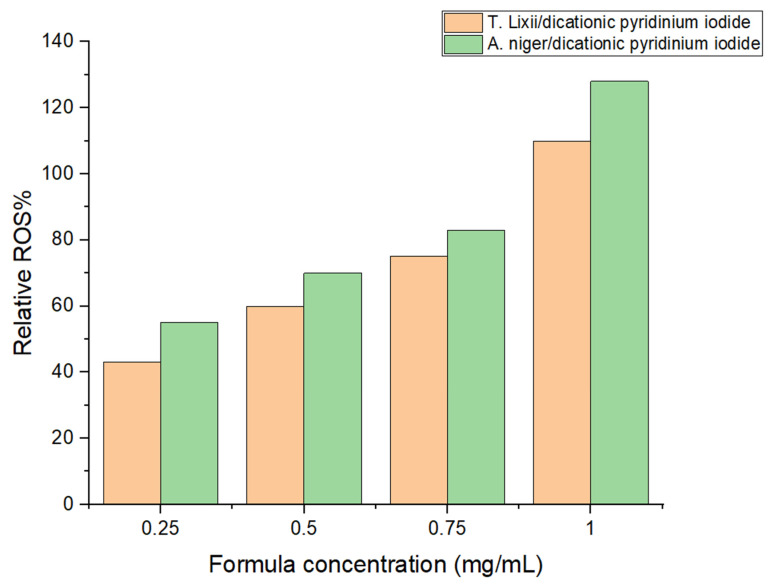
ROS production in the presence of the combined formulae.

Transmission electron microscopic (TEM) study was applied to the treated cells of *K. pneumoniae* against the combined drugs (*A. niger*/dicationic pyridinium iodide and *T. lixii*/dicationic pyridinium iodide). Figure 4 revealed a breakage in the cell wall and deformation of the cells, with leakage of the intracellular components that lead to cell death. Moreover, *A. niger*/dicationic pyridinium iodide and *T. lixii*/dicationic pyridinium iodide combined drugs showed potent antibacterial activity by inhibiting the bacterial growth after 6 and 4 h, respectively (Figure 5). Moreover, the reactive oxygen species (ROS) study of the treated bacterial cells revealed that by increasing the formula concentration the ROS increased, which elaborated the cell membrane damage and reduced bacterial cells’ viability (Figure 6).

Guo et al. [26] studied the antibacterial activity of *Aspergillus niger* crude extract (fraction B10) against *Agrobacterium tumefaciens* T-37 with an inhibition percentage of 98.22%, and the dose required to achieve 50% inhibition was 0.035 0.018 mg/mL. The antibacterial mechanism was evaluated by using electric conductivity, the release of proteins and nucleic acids, sodium dodecyl sulphate–polyacrylamide gel electrophoresis (SDS–PAGE), and the detection of reactive oxygen species (ROS). An increase in the relative electric conductivity of the supernatant was noticed with the addition of *Aspergillus niger* crude extract B10, which indicated that there was electrolyte transfer from the intracellular to the extracellular matrix. In contrast to the control group, the B10 fraction treated group showed a high increase in the amount of extracellular nucleic acid and protein between 0 and 18 h, and damage in the cytoplasmic membranes was noticed. SDS–PAGE analysis showed that the amounts of extracellular protein and nucleic acid were consistent with the lower levels of total protein inside the cells. It was demonstrated that B10 was responsible for the rise in ROS.

On the other hand, Qiao et al. [27] revealed that aspermerodione extracted from the endophytic fungus *Aspergillus* sp. TJ23 showed a synergistic effect with *β*-lactam antibiotics oxacillin and piperacillin as potent antibacterial combinations against MRSA. It was reported that combination therapy can be used as a promising strategy for combatting MRSA through extending the lifespan and efficacy of the currently employed antibiotics. The present investigation may pave the way into combating microbial infections through natural/synthetic combinations.

## 3. Materials and Methods

### 3.1. Tested Pathogens

*Pseudomonas aeruginosa*, *Acinetobacter baumannii*, *Proteus vulgaris*, *Staphylococcus aureus*, *Escherichia coli*, *Klebsiella aerogenes* and *Klebsiella pneumoniae* were kindly provided and identified by El-Shatby pediatric hospital using the Vitek 2 automated system (bioMerieux, Marcy l’Etoile, France) at the Medical Research Center, Faculty of Medicine, Alexandria University. The tested pathogens were kept in brain–heart infusion glycerol broth at −4 °C for further investigations, with monthly transfer into fresh media. The tested pathogens were identified as multi-drug resistant according to CLSI guidelines (Table S1).

### 3.2. Endophytic Fungal Isolation

Fungal samples were isolated from the roots of fully matured and healthy plants of *Mangifera Indica* at El Nubaria, Alexandria (30°41′57″ N, 30°40′1″ E), with firmed leaves and well-formed fruits, leaves and root systems. The root samples were rinsed with running tap water followed by deionized water and subsequently dipped in 70% ethanol (1–2 min), followed by sterilization in 0.1% sodium hypochlorite (2–3 min). They were further dipped in 70% ethanol and finally rinsed with distilled water. The roots were allowed to dry, and were cut aseptically into small pieces (1 cm^2^) and patched onto potato dextrose agar (PDA) (Himedia, Mumbai, India) plates containing streptomycin (SRL, Mumbai, India) at a concentration of 250 μg/mL to prevent bacterial contamination [28].

### 3.3. Molecular Identification of the Fungal Isolates

Fungal isolates were identified through ITS based DNA sequencing using the conserved ITS region of fungal gDNA amplified by general primers ITS4 (5′-TCCTCCGCTTATTGATATGC-3′) and ITS5 (5′-GGAAGTAAAAGTCGTAACAAGG-3′). ITS sequences of the identified fungi were submitted to GenBank for the retrieval of their accession numbers [28]. The study of percentage identity of the aligned sequences was carried out using a Kolmogorov–Smirnov statistical test in GeneDoc (version 2.7). Using the obtained sequences, phylogenetic analysis was performed and a phylogenetic tree was constructed through MEGA (v10.1.8) by the maximum likelihood Bootstrap (MLBS) method.

### 3.4. Seed Culture Preparations and Extraction of Fungal Secondary Metabolites

Spore suspension seed cultures were prepared according to CLSI guidelines [29]. Fungal isolates were inoculated (mycelial plugs (1 × 1 cm^2^)) into 300 mL potato-dextrose broth then incubated for 21 days at 25 °C under shaken conditions (140 rpm). At the end of the incubation period, the mycelia were harvested through filtration and the filtrate was extracted with chloroform/methanol (2:1, *v*/*v*) for 4 h. The crude fungal extract containing the bioactive compounds was stored at 4 °C for further experimental processes [28].

### 3.5. Antibacterial Activity of Fungal Secondary Metabolites

Antibacterial activity was carried out using the disc-diffusion method; the discs were saturated with 25 µL of each fungal extract (20 mg/mL) and placed on the surface of inoculated Müeller–Hinton agar plates [30]. Further antibacterial activity evaluation was carried out by assessing the minimal inhibitory concentration (MIC) and minimum bactericidal concentration (MBC) values [31]. MIC and MBC evaluations were performed through mixing 80 μL of sterile Müeller–Hinton broth, 20 μL tween 80, and 100 μL of the fungal secondary metabolites one at a time. The mixture was then diluted serially using a two-fold dilution in a 96-well microtiter plate. Then, 100 μL of 0.5 McFarland of the tested bacterial suspensions was inoculated in each well. MIC is the minimum concentration of the tested drugs that inhibited the bacterial growth, while the MBC is the minimum concentration needed to completely kill the microbial cells [32].

### 3.6. GC-MS Analysis of Fungal Secondary Metabolites

For the GC-MS analysis, 2 µL of samples were injected into the GC-MS device equipped with a spitless injector and a PE Auto system XL gas chromatograph interfaced with a Turbo-mass spectrometric mass selective detector system. The MS was operated in the EI mode (70 eV) with helium as the carrier gas (flow rate 1 mL/min) and an analytical column HP (length 30 mm−0.20 mm, 0.11 mm film thickness). The MS was operated in the total ion current (TIC) mode, scanning from *m*/*z* 30 to 400. The bioactive compounds were identified by comparing their retention time (RT in min) and mass spectra with the library of the National Institute of Standards and Technology (NIST), USA [33].

### 3.7. Chemical Synthesis of Dicationic Pyridinium Iodide Compounds

A mixture of 4-pyridinecarboxaldehyde (10 mmol) in ethanol (30 mL) and isonicotinic acid hydrazide (**1**) (10 mmol) with a few drops of hydrochloric acid was heated under reflux for 1 h. The solid obtained after the solvent evaporation under pressure was recrystallized from ethanol to furnish the desired Schiff base **2**.

**Scheme 1:**
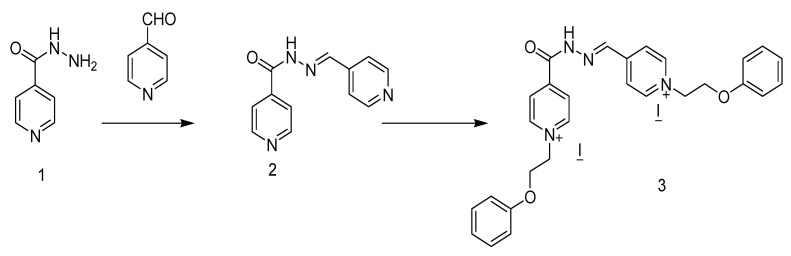
Chemical synthesis of dicationic pyridinium iodide compound.

The 4(2-iodoethoxy)benzene (10 mmol) was added under stirring to a solution of dipyridine Schiff base **2** (5 mmol) in acetonitrile (30 mL). Then, the reaction mixture was heated under reflux for 8 h, until the consumption of the starting material was indicated by TLC (silica gel, hexane-ethyl acetate). The solvent was reduced by evaporation under reduced pressure; the product formed was collected by filtration to afford the desired dicationic pyridinium iodide (**3**) (Scheme 1) [19].

#### 3.7.1. Characterization of the Prepared Dicationic Pyridinium Iodide (3)

mp: 82–83 °C. 1H NMR (400 MHz, DMSO-d6): δH = 4.54 (t, 4H, *J* = 8 Hz, 2 × NCH_2_), 5.18 (t, 4H, *J* = 8 Hz, 2 × OCH_2_), 6.94–6.96 (m, 6H, Ar-H), 7.31 (dd, 4H, *J* = 4 Hz, 8 Hz, Ar-H), 8.24 (d, 0.5H, *J* = 4 Hz, Ar-H), 8.33 (d, 0.5H, *J* = 4 Hz, Ar-H), 8.48 (d, 1.5H, *J* = 4 Hz, Ar-H), 8.63 (d, 1.5H, *J* = 4 Hz, Ar-H), 8.71 (s, 0.75H, H-C-N), 8.84 (s, 0.25H, H-C-N), 9.08 (d, 0.5H, *J* = 4 Hz, Ar-H), 9.22 (d, 1.5H, *J* = 4 Hz, Ar-H), 9.35 (d, 0.5H, *J* = 4 Hz, Ar-H), 9.45 (d, 1.5H, *J* = 4 Hz, Ar-H), 12.89 (s, 0.25H, CONH), 13.28 (s, 0.75H, CONH). 13C NMR (100 MHz, DMSO-d6): δC = 60.22, 60.90 (2 × NCH-), 66.43, 66.54 (2 × OCH-), 115.11, 121.94, 125.19, 126.76, 130.08, 144.95, 146.38, 147.05, 147.51, 149.69 (Ar-C), 157.93, 160.34 (C=N, C=O)

#### 3.7.2. Molecular Docking

The crystal structure of SHV-1 β-lactamase (Pdb: 3D4F), available at RCSB Protein Data Bank, was used as a template for constructing the 3D models [34].

##### Database Generation and Optimization

The ChemDraw application was used to draw the test compounds, and the MOE software database was utilized to gather these compounds once they had been drawn. Displaying hydrogen, computing partial charges, and using the default energy minimization were the three methods that were used in the optimization of the database. After the triangular matcher algorithm ligand was applied to the setting of the ligand placement, the default scoring function was employed to obtain the top five non-redundant poses that had the lowest binding energy of the test compound. In order to record the most effective potential molecular interactions, the docking of the optimized database was carried out using the induced fitting methodology. The docking score, expressed in Kcal/mol, was determined by combining the results of two different scoring functions—namely, alpha hydrogen bonding and London dG forces. The acquired results were organized into a list based on the S-scores that had an RMSD value of less than 2. The correctness of the employed software is heavily reliant on the training set, and the results of the molecular docking may be confirmed using a training set of experimental ligand–protein complexes. In order to guarantee a genuine and dependable docking strategy, the software that is being used needs to be able to reproduce the binding mode of an established reference inhibitor for the enzyme that is being targeted. The co-crystallized ligand LN1-255 was chosen as the comparison standard for the docking study in the experiment as a positive control (reference values). In the end, conformers that had the greatest binding scores and the best ligand–enzyme interactions were detected and examined [35].

### 3.8. Combination Study between the Fungal Extracts and the Synthesized Dicationic Pyridinium Iodide Compound

Combination studies were carried out according to White et al. [36]. The disc diffusion method was used to assess the possible differences in the inhibition zone diameter upon mixing the fungal extracts and the synthesized dicationic pyridinium iodide compound (1:1 *w*/*w*). Furthermore, the broth microdilution checkerboard technique was employed to study the synergistic effect between the fungal extract (Agent A) and dicationic pyridinium iodide compound (Agent B). Two-fold serial dilutions of the fungal extract and dicationic pyridinium iodide compound were dispensed in a 96-well microtiter plate with sub-MIC concentration. A 100 µL quantity of the bacterial suspension (1.5 × $10^{6}$ CFU/mL) was dispensed into each well and incubated for 24 h at 35 ± 2 °C. The fractional inhibitory concentration index (FICI) was computed, with the following equation:

FICI=FIC of agent A+FIC of agent B

where

$$\mathrm{FIC}\;\mathrm{of}\;\mathrm{agent}\;A=\frac{\mathrm{MIC}\;\mathrm{of}\;\mathrm{antimicrobial}\;\mathrm{agent}\;A\;\mathrm{in}\;\mathrm{combination}}{\mathrm{MIC}\;\mathrm{of}\;\mathrm{antimicrobial}\;\mathrm{agent}\;A\;\mathrm{alone}}$$

$$\mathrm{FIC}\;\mathrm{of}\;\mathrm{agent}\;B=\frac{\mathrm{MIC}\;\mathrm{of}\;\mathrm{antimicrobial}\;\mathrm{agent}\;B\;\mathrm{in}\;\mathrm{combination}}{\mathrm{MIC}\;\mathrm{of}\;\mathrm{antimicrobial}\;\mathrm{agent}\;B\;\mathrm{alone}}$$

FICI was considered as a synergistic when it was ≤0.5, and as additive when it was >0.5–1, indifferent when it was ≥1–4.0 and antagonistic when it was >4 [31].

### 3.9. Antibacterial Mechanism of Action of the Combined Formulae

#### 3.9.1. Transmission Electron Microscopic (TEM) Examination of the Treated Microbial Cells

On the basis of FIC and FICI values, the most susceptible bacterial strain (*K. pneumoniae*) was treated with the combined drugs. Samples were fixed using a universal electron microscope fixative. A series of dehydration steps were followed using ethanol and propylene oxide. The samples were then embedded in labeled beam capsules and polymerized. Thin sections of cells exposed to extracts were cut using LKB 2209-180 ultra-microtome and stained with a saturated solution of uranyl acetate for half an hour and lead acetate for 2 min [31]. Electron Micrographs were taken using a Transmission Electron Microscope (JEM-100 CX Joel).

#### 3.9.2. Time-Kill Curve

A time-kill curve was investigated to estimate the optimum time required to inhibit the bacterial vegetative cells. Fungal secondary metabolites combined with the dicationic pyridinium iodide compound (FIC and FICI values of each) were added one at a time to 10 mL Müeller–Hinton broth containing 1 × 10^6^ CFU/mL bacterial cells. Aliquots were withdrawn to assess the bacterial growth through different incubation time (0, 2, 4, 6, 8, 12 and 24 h) at OD 600 nm [37].

#### 3.9.3. Reactive Oxygen Species (ROS) Study

The reactive oxygen species (ROS) generation assay was measured according to Almotairy et al. [38] and Bhuvaneshwari et al. [39] using 2,7-dichlorofluorescin diacetate (DCFH-DA) dye by comparing the extracellular ROS of the treated and control bacterial cells.

## 4. Conclusions

In the current study, two endophytic fungal strains with antimicrobial activities were isolated from *Mangifera Indica* roots and identified as *Aspergillus niger* MT597434.1 and *Trichoderma lixii* KU324798.1. A dicationic pyridinium iodide compound was synthesized and then evaluated for its potential synergistic effect with the extracted fungal crude extract. The molecular modeling study revealed that the synthesized dicationic pyridinium iodide compound and the extracted fungal secondary metabolites showed promising inhibitory effects against the SHV-1 enzyme. The combination of *A. niger* and *T. lixii* secondary metabolites with the dicationic pyridinium iodide compound showed a synergistic effect against *K. pneumoniae*. Fungal secondary metabolites combined drugs inhibited the bacterial growth after 6 and 4 h through cell wall breakage and cells’ deformation with intracellular components leakage and increased ROS production, which led to bacterial cell death. This study proved the importance of the combination of fungal secondary metabolites and some synthetic drugs against multi-drug resistant microbial cells through several modes of action, which may pave the way to more available naturally derived options.

## Figures and Tables

**Table 1:** Antibacterial activity of fungal bioactive secondary metabolites against MDR pathogens.

Tested Pathogens	*T. lixii* Crude Extract			*A. niger* Crude Extract		
	IZ Diameter (mm)	MIC (µg/mL)	MBC (µg/mL)	IZ Diameter (mm)	MIC (µg/mL)	MBC (µg/mL)
*Pseudomonas aeruginosa*	14.5	250	500	19.0	250	500
*Acinetobacter baumannii*	9.0	500	1000	7.5	500	1000
*Proteus vulgaris*	12.5	250	500	13.0	250	500
*Staphylococcus aureus*	11.0	500	1000	21.0	250	1000
*Escherichia coli*	20.0	250	250	12.0	250	250
*Klebsiella aerogenes*	8.0	500	1000	7.5	1000	1000
*Klebsiella pneumoniae*	16.0	250	500	11.0	250	500

**Table 2:** Retention time (RT) and probable compounds according to MS library.

Strain	RT	Compound	Area%
*A. niger*	7.29	1-Octanol, 2,7-dimethyl	6.72
	19.15	2-(Octadecyloxy)ethanol	3.00
	12.74	Pentadecanoic acid, 14-methyl-, methyl ester	26.66
	14.17	9-Octadecenoic acid (*Z*)-, methyl ester	18.01
*T. lixii*	7.49	2,4-Decadienal, (*E*,*E*)	10.69
	13.36	Undecanoic acid	7.98
	14.59	9-Octadecenoic acid (*Z*)-, methyl ester	10.32
	16.65	1,2-15,16-Diepoxyhexadecane	7.84
	18.42	Heptadecane, 9hexyl	1.86

**Table 3:** Binding scores (Kcal/mol) of the investigated molecule with the target SHV-1 enzymes.

	Compounds	Docking Score (kcal/mol)	2D Binding Mode within SHV-1 Active Site
Ref.	Co-crystallized ligand LN1-255	−6.44	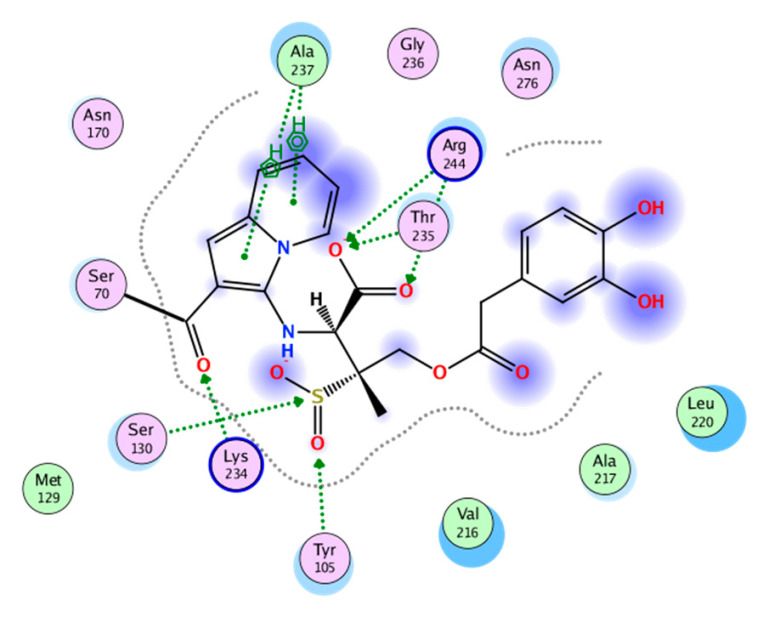
Syn. Comp.	Dicationic pyridinium iodide compound	−6.38	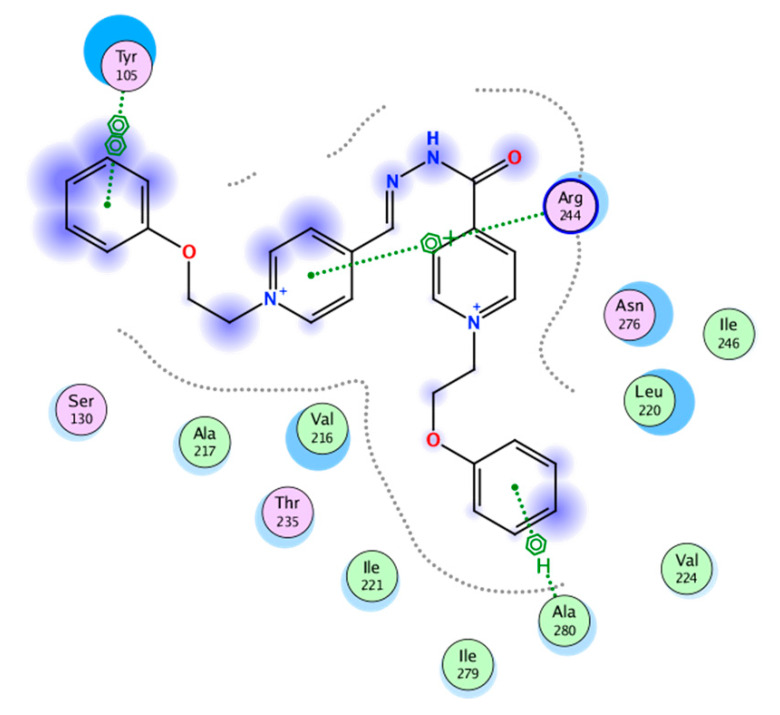
*A. niger*	1-Octanol, 2,7-dimethyl	−5.00	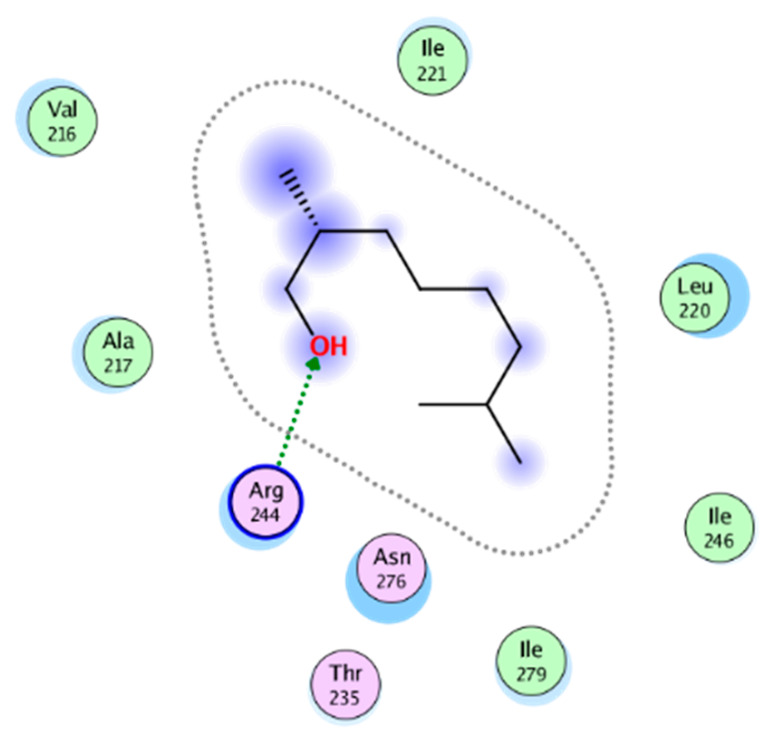
	2-(Octadecyloxy)ethanol	−6.40	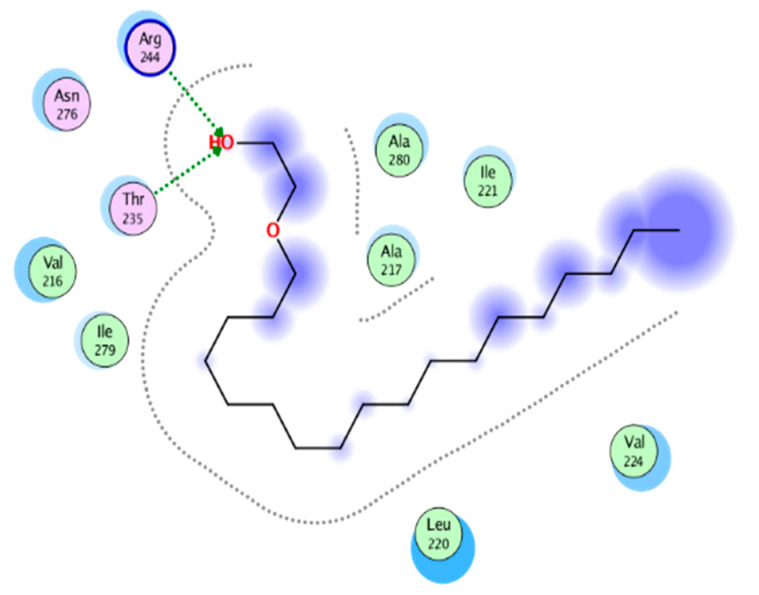
	Pentadecanoic acid, 14-methyl-, methyl ester	−6.51	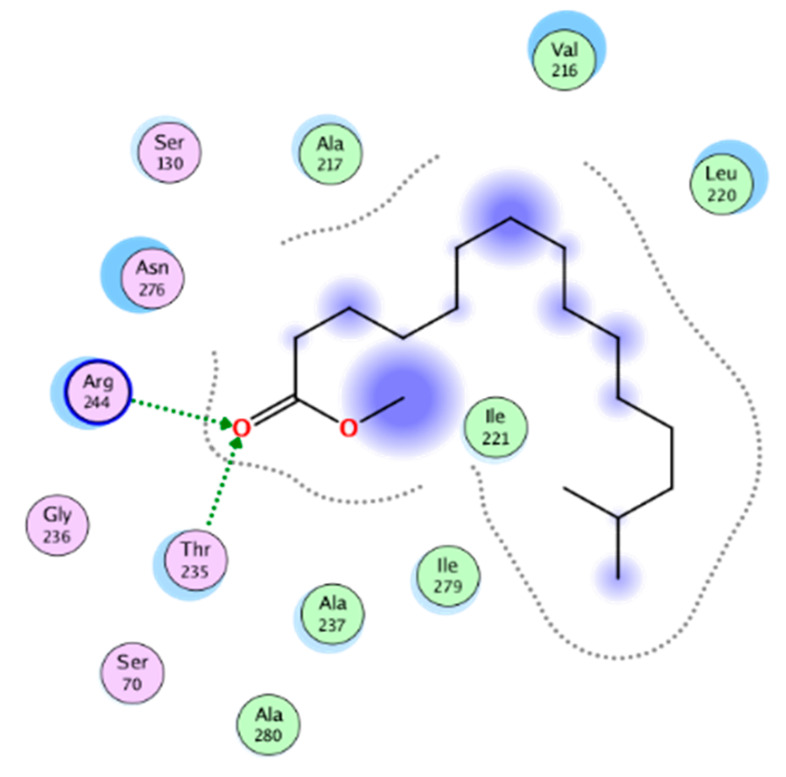
	9-Octadecenoic acid (*Z*)-, methyl ester	−6.50	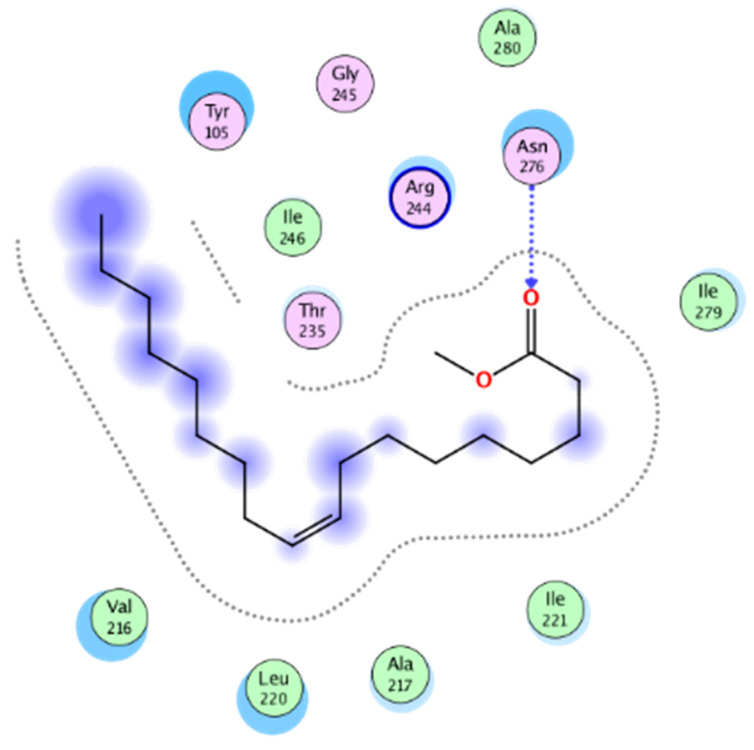
*T. lixii*	2,4-Decadienal, (*E*,*E*)	−5.04	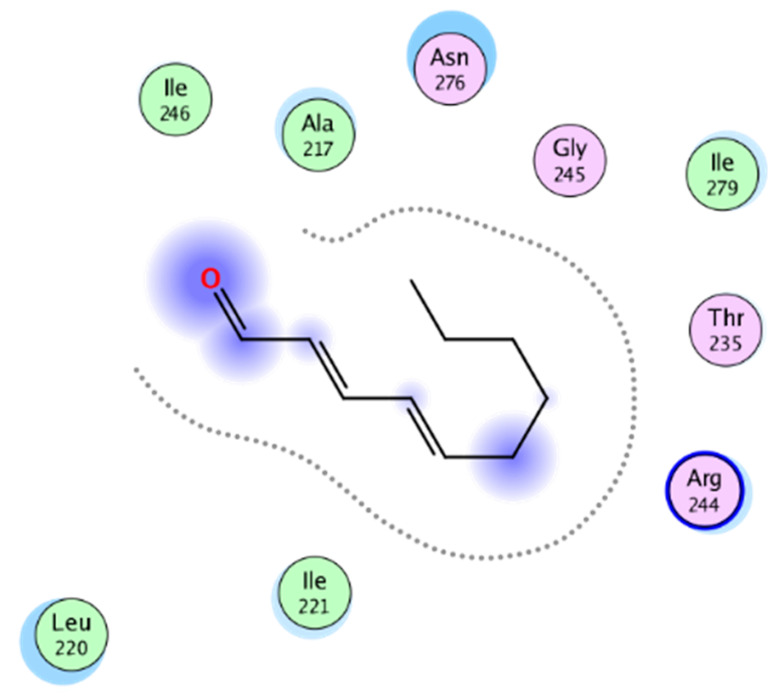
	Undecanoic acid	−5.62	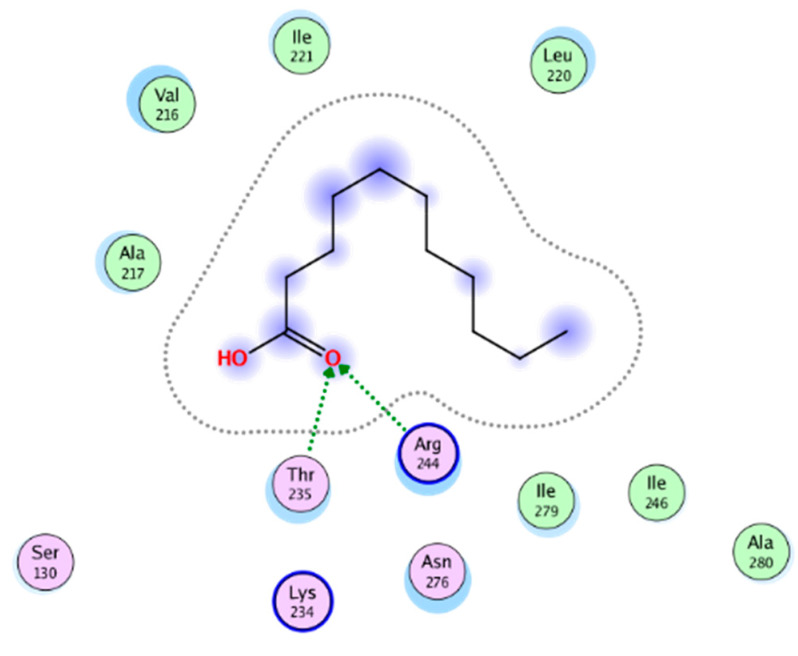
	9-Octadecenoic acid (*Z*)-, methyl ester	−6.96	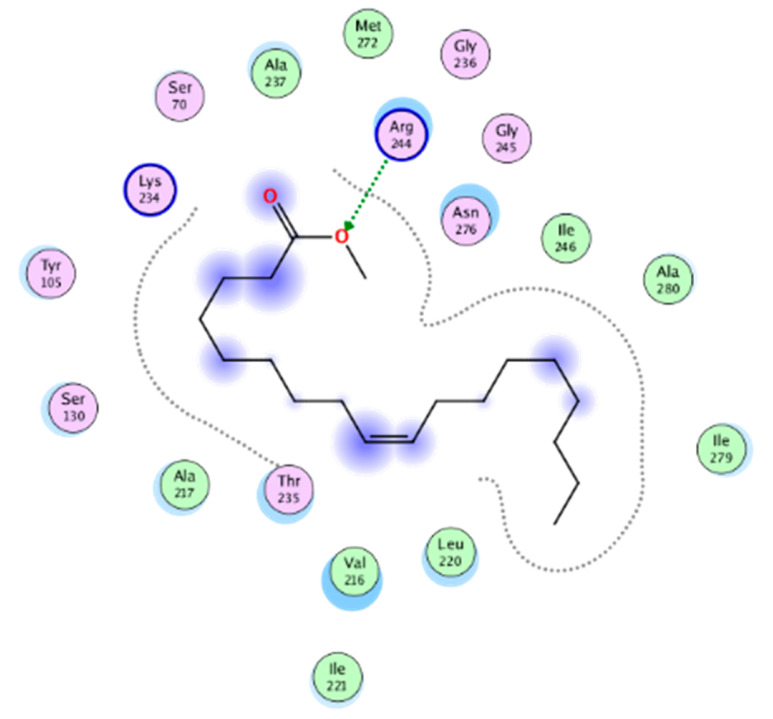
	1,2-15,16-Diepoxyhexadecane	−6.56	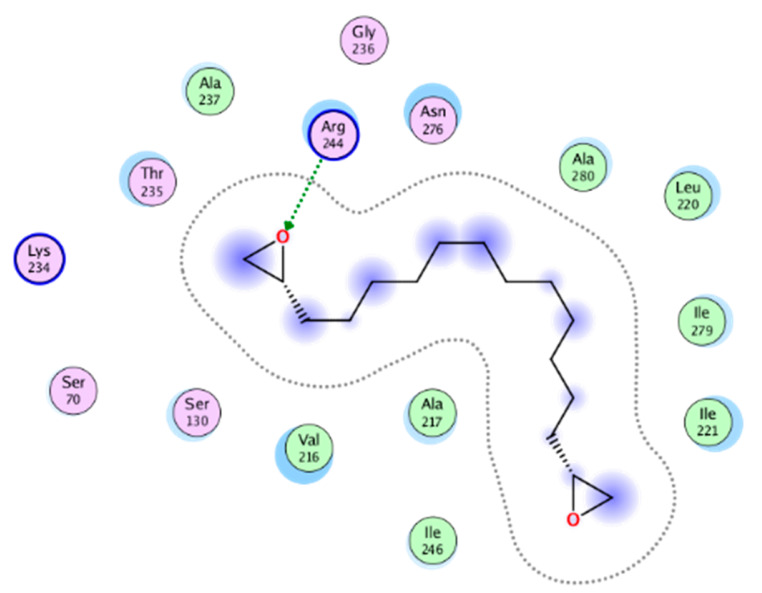
	Heptadecane, 9hexyl	−6.99	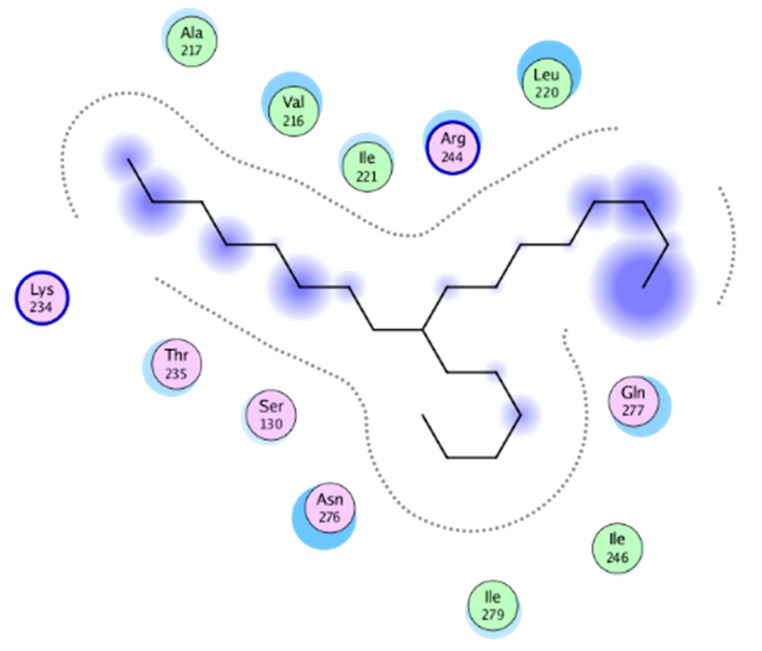

**Table 4:** Sensitivity test of *T. lixii* and *A. niger* crude extract in combination with dicationic pyridinium iodide compound.

Tested Pathogens	IZ Diameter (mm)				
	Dicationic Pyridinium Iodide	*A. niger* Crude Extract	*T. lixii* Crude Extract	*A. niger* Crude Extract + Dicationic Pyridinium Iodide	*T. lixii* Crude Extract + Dicationic Pyridinium Iodide
*P. aeruginosa*	10.0	19.0	14.5	30.5	14.5
*A. baumannii*	9.0	7.5	9.0	17.0	9.0
*P. vulgaris*	10.5	13.0	12.5	24.5	12.5
*S. aureus*	14.3	21.0	11.0	22.0	16.0
*E. coli*	13.0	12.0	20.0	34.5	20.0
*K. aerogenes*	14.0	7.5	8.0	23.0	8.0
*K. pneumoniae*	12.0	11.0	16.0	24.5	32.0

**Table 5:** Synergy test of *T. lixii* crude extract and *A. niger* crude extract in combination with the dicationic pyridinium iodide compound.

Antibacterial Agent		MIC of Single Drug (µg/mL)	MIC of Combined Drug (µg/mL)	FIC	FICI	Interpretation
*K. pneumoniae*	*dicationic pyridinium iodide*	250.0	50.0	0.20	-	-
	*A. niger* crude extract	250.0	37.5	0.15	0.35	Synergy
	*T. lixii* crude extract	250.0	50.0	0.20	0.4	Synergy

-: Not detected.

## Data Availability

The datasets analyzed during the current study are available from the National Center for Biotechnology Information (NCBI) database (accessed on December 2021).

## Supplementary Materials

The following supporting information can be downloaded at: https://www.mdpi.com/article/10.3390/molecules28062434/s1, Table S1: The resistance prevalence in the tested pathogens.

## Sample Availability

Not Available.
