# Supplementary material for: Fungal Secondary Metabolites/Dicationic Pyridinium Iodide Combinations in Combat against Multi-Drug Resistant Microorganisms
Source: Molecules. 2023 Mar 7;28(6):2434. doi: 10.3390/molecules28062434 (PMC10058977; doi:10.3390/molecules28062434)
Supplement: Supplementary file 1 [file molecules-28-02434-s001.zip › molecules-2222591-supplementary.pdf]

Fungal Secondary Metabolites/Dicationic Pyridinium Iodide Combinations in Combat against Multi-Drug Resistant Microorganisms

Ayoub M. Abdelalatif [1], Bassma H. Elwakil [2,*], Mohamed Zakaria Mohamed [3], Mohamed Hagar [3] and Zakia A. Olama [1]

[1] Department of Botany & Microbiology, Faculty of Science, Alexandria University, Alexandria 21568, Egypt; ayoub.alhallaj@gmail.com (A.M.A.); zakiaolama52@gmail.com (Z.A.O.)
[2] Department of Medical Laboratory Technology, Faculty of Applied Health Sciences Technology, Pharos University in Alexandria, Alexandria 21526, Egypt
[3] Department of Chemistry, Faculty of Science, Alexandria University, Alexandria 21568, Egypt; mohamed.zakaria@alexu.edu.eg (M.Z.M.); mohamedhaggar@gmail.com (M.H.)
* Correspondence: bassma.hassan@pua.edu.eg

Supplementary Table

Table S1. The resistance prevalence in the tested pathogens

Tested pathogens	IZ Diameter (mm)							
	Ampicillin	Cefazolin	Amikacin	Pipracillin	Amoxacillin/ Clavulanate	Ampicillin/ Sulbactam	R*	S*
Pseudomonas aeruginosa	10.0 ± 0.7	16.0 ± 0.2	10.0 ± 0.9	19.0	22.0 ± 0.8	11.0 ± 0.6	>17	>23
Acinetobacter baumannii	11.0 ± 0.5	15.0 ± 0.7	11.0 ± 0.3	7.5	15.0 ± 0.3	11.0 ± 0.9	<13	<19
Proteus vulgaris	11.0 ± 0.4	11.0 ± 0.7	11.0 ± 0.3	13.0	13.0 ± 0.9	21.0 ± 0.6	>17	>23
Staphylococcus aureus	12.0 ± 0.7	16.0 ± 0.4	13.0 ± 0.5	21.0	20.0 ± 0.5	20.0 ± 0.3	<13	<19
Escherichia coli	18.0 ± 0.5	16.0 ± 0.2	12.0 ± 0.8	12.0	16.0 ± 0.3	11.0 ±. 0.2	>17	>23
Klebsiella aerogenes	11.0 ± 0.9	17.0 ± 0.6	18.0 ± 1.0	7.5	15.0 ± 0.5	19.0 ± 0.7	<13	<19
Klebsiella pneumoniae	11.0 ± 0.2	16.0 ± 0.9	13.0 ± 0.4	11.0	23.0 ± 0.4	11.0 ± 0.5	>17	>23

* According to CLSI (2015), R: Resistant, S: Sensitive
